# A rare case of penile metastasis from renal cell carcinoma following combination therapy with immune checkpoint and tyrosine-kinase inhibitors: A case report and literature review

**DOI:** 10.1097/MD.0000000000043622

**Published:** 2025-07-25

**Authors:** Takumi Arai, Daisuke Obinata, Kazuki Ohashi, Yuki Inagaki, Sho Hashimoto, Ken Nakahara, Tsuyoshi Yoshizawa, Junichi Mochida, Satoru Takahashi

**Affiliations:** a Department of Urology, Nihon University School of Medicine, Tokyo, Japan.

**Keywords:** malignant priapism, penile metastasis, renal cell carcinoma

## Abstract

**Rationale:**

Penile metastasis from renal cell carcinoma (RCC) is an extremely rare clinical entity, particularly in the era of immune-checkpoint inhibitor and tyrosine-kinase inhibitor combination therapy. The mechanisms, clinical presentation, and optimal management of such cases remain poorly understood.

**Patient concerns:**

A 75-year-old man with metastatic RCC undergoing systemic therapy developed persistent, painful erections (malignant priapism), along with urinary retention, general malaise, and back pain, 2 years after initiating treatment.

**Diagnosis:**

Magnetic resonance imaging of the penis revealed a hypointense lesion on T2-weighted imaging and restricted diffusion on diffusion-weighted imaging, suggestive of tumor infiltration into the corpus cavernosum. Blood gas analysis from corporal aspiration was consistent with nonischemic priapism. A diagnosis of penile metastasis from RCC was established.

**Interventions:**

Embolization of the common penile artery was performed, followed by palliative radiotherapy (30 gray in 10 fractions).

**Outcomes:**

Despite these interventions, penile rigidity persisted, though partial symptomatic relief and pain reduction were achieved. Disease progression was noted, and the patient died approximately 3 months after the diagnosis of penile metastasis.

**Lessons:**

This is the first reported case of penile metastasis from RCC during immune-checkpoint inhibitor-tyrosine-kinase inhibitor therapy, highlighting a rare but clinically important metastatic pattern potentially unmasked by prolonged survival. Retrograde dissemination via Batson venous plexus may underlie this presentation. Radiotherapy and embolization may offer partial symptomatic relief, but the prognosis remains poor. Accumulation of further cases is necessary to guide future management strategies.

## 1. Introduction

Renal cell carcinoma (RCC) is a common urologic malignancy. In advanced stages, it frequently metastasizes to multiple organs, including the lungs, bones, liver, brain, and adrenal gland. However, metastasis of renal carcinoma to the penis is rare, and its clinical presentation, diagnostic approaches, and treatment strategies are not well established.^[1]^

To our knowledge, this is the first reported case of penile metastasis in a patient with RCC receiving combination therapy with an immune-checkpoint inhibitor (ICI) and a tyrosine-kinase inhibitor (TKI). This case underscores the importance of recognizing unusual metastatic patterns in the era of ICI therapy.

Penile metastasis can also be seen in advanced cancers other than renal cancer, with common symptoms including malignant priapism, penile enlargement, pain, and dysuria.^[2,3]^ Although vascular embolization and radiation therapy can alleviate the symptoms of malignant priapism, they are not curative treatments, and treatment strategies must consider the quality of life of patients.

In this report, we present a case of penile metastasis of RCC diagnosed as a result of malignant priapism.

## 2. Case report

**Figure 1.**
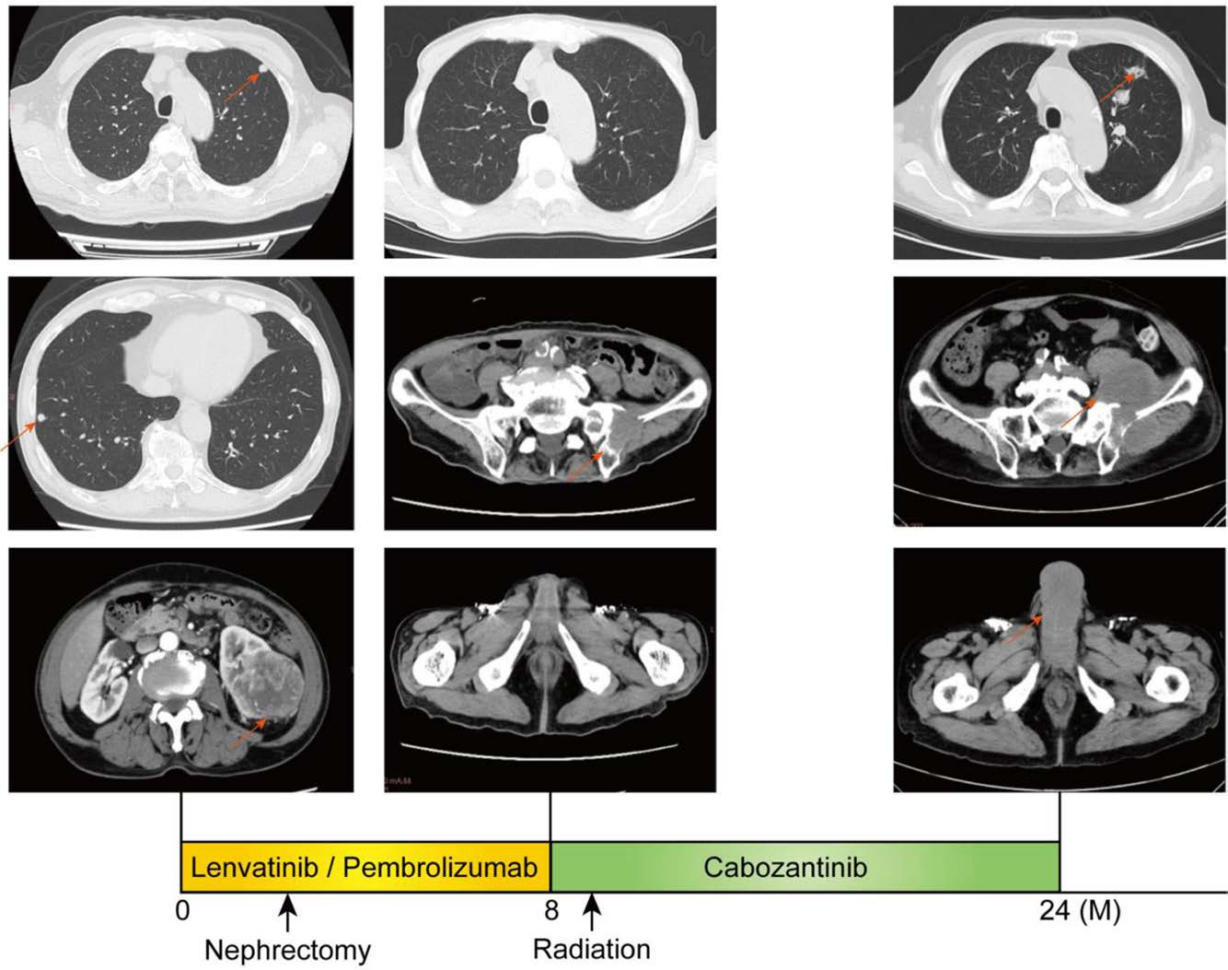
Clinical course following renal cell carcinoma diagnosis. Representative imaging findings at each time point are presented.

**Figure 2.**
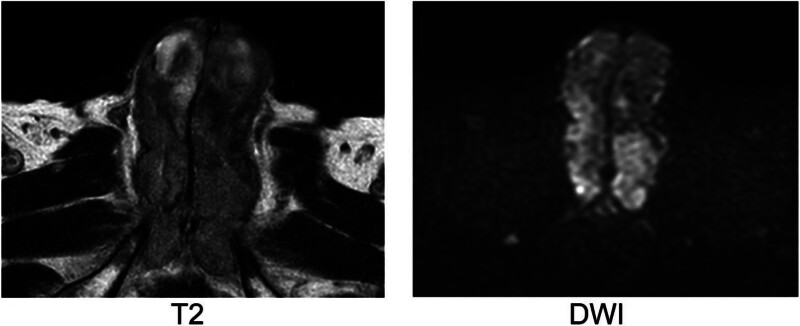
Magnetic resonance imaging of the penis at the time of persistent priapism. T2 shows a hypointense area, and DWI demonstrates restricted diffusion. DWI = diffusion-weighted imaging, T2 = T2-weighted imaging.

A 75-year-old man with a history of hypertension was diagnosed with metastatic RCC following a computed tomography scan that showed a tumor in the lower pole of the left kidney with contrast enhancement and a pulmonary nodule suggestive of metastasis. After initiating combination therapy with lenvatinib and pembrolizumab, the patient underwent a left nephrectomy following confirmation of tumor shrinkage. The pathological diagnosis was clear cell carcinoma, stage T3a. Eight months after the start of treatment, a new left iliac metastasis appeared, and the patient was switched from lenvatinib and pembrolizumab to cabozantinib along with radiation therapy and denosumab (Fig. 1). Twenty-five months after treatment initiation, the patient developed urinary retention, general malaise, back pain, and malignant priapism with mild pain. Penile cavernosal puncture and blood gas analysis revealed a PaO_2_ and PaCO_2_ of 97.2 and 32.1 mm Hg, respectively, leading to a diagnosis of nonischemic persistent erection. Despite the cavernosal puncture, the patient continued to experience malignant priapism. Magnetic resonance imaging showed low-signal areas on T2-weighted images (T2) and high-signal areas on diffusion-weighted images in the penile cavernous body, leading to the diagnosis of malignant priapism due to a metastatic penile tumor (Fig. 2). The patient underwent embolization of the common penile artery, and although penile hardness temporarily decreased, erection was sustained. Subsequently, the patient received irradiation of 30 gray (Gy) delivered in 10 fractions to the penile region for symptomatic palliation. Although a further decrease in penile hardness and reduction in pain were observed, progression of the disease, mainly iliac metastasis and lung metastasis, was observed, and the patient died approximately 3 months after the onset of penile metastasis.

## 3. Discussion and literature review

Priapism is a condition caused by the abnormal regulation of blood flow to the penis and can be broadly classified into ischemic (low-flow) and nonischemic (high-flow) types. In general, malignancy-associated priapism is typically ischemic in nature, resulting from direct tumor invasion of the venous system, which impairs venous outflow. However, in the present case, blood gas analysis and magnetic resonance imaging of the penile corpus cavernosum revealed findings that were inconsistent with ischemic priapism. Generally, nonischemic priapism in the setting of malignancy may occur due to altered vascular dynamics caused by tumor involvement.^[3]^

Metastatic penile tumors are extremely rare, with malignancies of the genitourinary system and pelvic organs such as the rectum and sigmoid colon accounting for 75% of primary sites.^[4]^ Among urological malignancies, the most common primary sites of penile metastasis are the bladder and prostate, while RCC accounts for only approximately 7% of all cases.^[3]^ The proposed mechanisms of penile metastasis include hematogenous and lymphatic spread. Additionally, retrograde venous dissemination via the valveless Batson plexus has been reported as a unique pathway.^[5]^ In this route, tumor cells are thought to reach the penis through the lumbar venous plexus, which lacks the venous valves.

Batson plexus, first described in 1940, is a valveless, interconnected venous network that links the deep pelvic and thoracic veins with the internal vertebral venous plexus.^[6]^ This system permits pressure-driven, bidirectional venous flow and functions as a collateral pathway during conditions of increased intra-abdominal pressure or venous congestion.^[6]^ These anatomical characteristics provide a plausible retrograde route for tumor cell dissemination from pelvic or abdominal organs to distal sites, such as the penis.^[7]^

In the present case, metastasis to the left iliac bone preceded the appearance of penile metastasis from left-sided RCC. We hypothesize that treatment with TKI, which exerts anti-angiogenic effects, may have contributed to retrograde dissemination. This could result from TKI-induced vascular remodeling and capillary rarefaction,^[8]^ leading to increased peripheral vascular resistance and potentially elevated venous pressure. Such hemodynamic alterations might impair venous return and promote retrograde tumor cell dissemination through valveless venous networks, such as Batson plexus. Furthermore, localized venous congestion caused by iliac bone metastasis might have facilitated retrograde tumor spread into the dorsal penile venous system.

Management of malignant priapism requires the identification of the underlying etiology and appropriate therapeutic decision-making. In cases of nonischemic priapism, such as the current case, standard interventions such as corporal aspiration or α-adrenergic agonists are generally ineffective. In such instances, vascular embolization or radiotherapy may be considered palliative treatment options.^[9]^

In our case, the priapism was nonischemic in nature, and arterial embolization of the common penile artery was initially undertaken to rapidly alleviate symptoms by reducing arterial inflow. In addition, angiography performed during embolization allowed for assessment of a possible arteriovenous fistula, a known cause of malignant priapism secondary to tumor infiltration.^[10]^ Although no definitive arteriovenous fistula was detected, tumor-associated hypervascularity and venous congestion were considered likely contributors to the persistent erection. As embolization of the common penile artery was performed initially, but it did not fully resolve the priapism, palliative radiotherapy (30 Gy in 10 fractions) was administered, which resulted in pain relief and reduced penile rigidity. Consistent with prior reports,^[9]^ this case further supports the utility of radiotherapy as a palliative option for nonischemic priapism caused by metastatic penile tumors.

The prognosis of patients with penile metastasis from RCC is extremely poor,^[5]^ particularly in those presenting with malignant priapism.^[9]^ This poor prognosis may be attributed to the fact that penile metastases are rarely isolated; rather, they often occur in the context of disseminated disease.^[11]^ In our case, progression of pulmonary and iliac metastases was observed at the time of priapism onset, accompanied by a significant deterioration of the general condition of the patient.

To the best of our knowledge, this is the first reported case of penile metastasis in a patient with RCC treated with a combination of ICI and TKI therapy. Specifically, no prior cases have described penile metastasis occurring during cabozantinib administration following cytoreductive nephrectomy following ICI-TKI combination therapy.

A PubMed search using the keywords “metastatic renal cell carcinoma” and “penis,” limited to publications after 2017 – the year ICIs were first approved for RCC – identified only 6 reported cases of penile metastases from RCC with clearly described treatment details (excluding the present case). All of these involved TKI monotherapy (e.g., sunitinib or lenvatinib) as first-line treatment for metastatic disease. In one case, ICI was administered as adjuvant therapy following surgery. To date, no reports have described penile metastasis in patients receiving ICI-TKI combination therapy (Table 1).^[1,12–16]^

**Table 1:** Summary of reported cases of penile metastasis from renal cell carcinoma after the introduction of immune-checkpoint inhibitors.

No	Author	Age	Penile symptoms	Tumor side	Pathology	Metastatic sites at diagnosis	Treatment	Treatment for penile lesion	Outcome
1	Zhu^[12]^	38	Penile pain	Unknown	Clear cell carcinoma	Brain, lungs, adrenal glands	Surgery, TKI following ICI	None	Died at 2 d
2	Safriadi^[13]^	69	Hard mass	Left	Papillary	Penis, testis	TKI, Surgery	Total penectomy	Died at 6 mo
3	Joshi^[14]^	66	Dysuria	Left	Clear cell carcinoma	Adrenal, lung, bone	Surgery, TKI	None	Unknown
4	Luo^[15]^	60	Persistent priapism	Left	Clear cell carcinoma	None	Surgery, TKI	Radiotherapy	Died at 3 mo
5	Cho^[1]^	75	Penile pain and swelling	Right	Clear cell carcinoma	None	Surgery, TKI	Surgery, radiotherapy	Unknown
6	Khalil^[16]^	51	Penile pain and irradiation	Right	Clear cell carcinoma	None	Surgery, adjuvant ICI	None	Unknown
7	Present case	75	Persistent priapism	Left	Clear cell carcinoma	Lung, bone	Surgery, ICI-TKI	Radiotherapy	Died at 3 mo

ICI = immune-checkpoint inhibitor, TKI = tyrosine-kinase inhibitor.

Although penile metastasis from RCC remains exceedingly rare, the extended survival afforded by ICI-TKI therapies may reveal metastatic patterns not previously encountered, including the involvement of anatomically unusual sites, such as the penis. Recent literature has reported the emergence of atypical metastatic sites in mRCC following ICI-TKI therapy, including the adrenal glands, brain, nasal ala skin, and choroid.^[17–19]^ Among these, choroidal metastasis – though extremely rare – is particularly significant due to the risk of irreversible visual loss if not promptly diagnosed and managed.^[18]^ These findings reflect evolving metastatic patterns in the era of prolonged survival and immune modulation, underscoring the importance of maintaining a high index of suspicion for nonclassical metastatic presentations in patients receiving combination systemic therapies.

In the terminal phase, a direct account from the patient was not feasible due to impaired consciousness. However, according to his family, he had experienced considerable distress from urinary difficulties related to priapism while at home, which they found emotionally challenging to witness. As his condition deteriorated, they came to understand that these symptoms were manifestations of systemic decline and acknowledged the importance of palliative care. They expressed relief when symptom management measures – such as urethral catheterization – alleviated his discomfort and appeared to soften his facial expression.

## 4. Conclusion

This case presents a rare instance of malignant priapism caused by metastatic RCC. Retrograde spread via Batson venous plexus is considered a possible mechanism for penile metastasis. Vascular embolization and radiotherapy provide partial symptomatic relief for the management of priapism. The accumulation and detailed reporting of further cases are essential to establish optimal treatment strategies for metastatic penile tumors.

## Acknowledgments

We would like to thank Editage (www.editage.com) for the English language editing.

## Author contributions

**Conceptualization:** Takumi Arai, Daisuke Obinata.

**Data curation:** Kazuki Ohashi, Yuki Inagaki, Sho Hashimoto.

**Investigation:** Daisuke Obinata, Ken Nakahara, Tsuyoshi Yoshizawa, Junichi Mochida.

**Supervision:** Daisuke Obinata, Satoru Takahashi.

**Writing – original draft:** Takumi Arai, Daisuke Obinata.

**Writing – review & editing:** Satoru Takahashi.
